# Novel prognostic scoring systems for severe CRS and ICANS after anti-CD19 CAR T cells in large B-cell lymphoma

**DOI:** 10.1186/s13045-024-01579-w

**Published:** 2024-08-06

**Authors:** Pierre Sesques, Amy A. Kirkwood, Mi Kwon, Kai Rejeski, Michael D. Jain, Roberta Di Blasi, Gabriel Brisou, François-Xavier Gros, Fabien le Bras, Pierre Bories, Sylvain Choquet, Marie-Thérèse Rubio, Gloria Iacoboni, Maeve O’Reilly, René-Olivier Casasnovas, Jacques-Olivier Bay, Mohamad Mohty, Magalie Joris, Julie Abraham, Cristina Castilla Llorente, Mickael Loschi, Sylvain Carras, Adrien Chauchet, Laurianne Drieu La Rochelle, Olivier Hermine, Stéphanie Guidez, Pascale Cony-Makhoul, Patrick Fogarty, Steven Le Gouill, Franck Morschhauser, Thomas Gastinne, Guillaume Cartron, Marion Subklewe, Frederick L. Locke, Robin Sanderson, Pere Barba, Roch Houot, Emmanuel Bachy

**Affiliations:** 1 Hematology Department, Hospices Civils de Lyon, 165 Chemin du Grand Revoyet, 69410 Pierre Bénite, Lyon, France; 2 Cancer Research UK & UCL Cancer Trials Centre, UCL Cancer Institute, University College London, London, UK; 3 Department of Hematology, Hospital General Universitario Gregorio Marañón, Madrid, Spain; 4 Department of Medicine III – Hematology/Oncology, LMU University Hospital, LMU Munich, Munich, Germany; 5 Department of Blood and Marrow Transplant and Cellular Immunotherapy, Moffitt Cancer Center, Tampa, USA; 6 Hematology Department, Hôpital Saint Louis, Paris, France; 7 Hematology Department, Institut Paoli Calmettes, Marseille, France; 8 Hematology Department, CHU de Bordeaux, Bordeaux, France; 9 Hematology Department, Hôpital Henri Mondor, Créteil, France; 10 Hematology Department, CHU de Toulouse, Toulouse, France; 11 Hematology Department, Hôpital de la Pitié Salpêtrière and AP-HP Sorbonne Université, Paris, France; 12 Hematology Department, CNRS UMR 7365, CHRU de Nancy, Nancy, France; 13 Department of Hematology, University Hospital Vall d’Hebron, Barcelona, Spain; 14 Experimental Hematology, Vall d’Hebron Institute of Oncology (VHIO), Barcelona, Spain; 15 Department of Haematology, University College London Hospitals, London, UK; 16 Hematology Department, CHU de Dijon and INSERM 1231, Dijon, France; 17 Hematology Department, CHU de Clermont Ferrand, Clermont-Ferrand, France; 18 Hematology Department, Hôpital Saint Antoine, Inserm UMRs 938, Sorbonne University, Paris, France; 19 Hematology Department, CHU d’Amiens, Amiens, France; 20 Hematology Department, CHU de Limoges, Limoges, France; 21 Hematology Department, Gustave Roussy Cancer Campus, Villejuif, Paris, France; 22 Hematology Department, CHU de Nice, Nice, France; 23 Hematology Department, Institute for Advanced Biosciences (INSERM U1209, CNRS UMR 5309), CHU de Grenoble and University Grenoble-Alpes, La Tronche, France; 24 Hematology Department, CHU de Besançon, Besançon, France; 25 Hematology Department, CHU de Tours, Tours, France; 26 Hematology Department, Hôpital Necker, Paris, France; 27 Hematology Department, CHU de Poitiers, Poitiers, France; 28 Medical and Scientific Affairs Department, LYSARC, Lyon, France; 29 Biostatistics Department, LYSARC, Lyon, France; 30 Hematology Department, Institut Curie, Paris, France; 31 Hematology Department, CHU de Lille, Lille, France; 32 ULR 7365 - GRITA - Groupe de Recherche sur les formes Injectables et les Technologies Associées, Lille University, Lille, France; 33 Hematology Department, CHU de Nantes, Nantes, France; 34 Hematology Department, CHU de Montpellier and UMR-CNRS, Montpellier, France; 35 Department of Haematology, King’s College Hospital, London, UK; 36 Hematology Department, CHU de Rennes, Rennes, France; 37 Lymphoma Immuno-Biology, CIRI, Inserm U1111, Lyon, France

## Abstract

Autologous anti-CD19 chimeric antigen receptor (CAR) T cells are now used in routine practice for relapsed/refractory (R/R) large B-cell lymphoma (LBCL). Severe (grade ≥ 3) cytokine release syndrome (CRS) and immune effector cell-associated neurotoxicity (ICANS) are still the most concerning acute toxicities leading to frequent intensive care unit (ICU) admission, prolonging hospitalization, and adding significant cost to treatment. We report on the incidence of CRS and ICANS and the outcomes in a large cohort of 925 patients with LBCL treated with axicabtagene ciloleucel (axi-cel) or tisagenlecleucel (tisa-cel) in France based on patient data captured through the DESCAR-T registry. CRS of any grade occurred in 778 patients (84.1%), with 74 patients (8.0%) with grade 3 CRS or higher, while ICANS of any grade occurred in 375 patients (40.5%), with 112 patients (12.1%) with grade ≥ 3 ICANS. Based on the parameters selected by multivariable analyses, two independent prognostic scoring systems (PSS) were derived, one for grade ≥ 3 CRS and one for grade ≥ 3 ICANS. CRS-PSS included bulky disease, a platelet count < 150 G/L, a C-reactive protein (CRP) level > 30 mg/L and no bridging therapy or stable or progressive disease (SD/PD) after bridging. Patients with a CRS-PSS score > 2 had significantly higher risk to develop grade ≥ 3 CRS. ICANS-PSS included female sex, low level of platelets (< 150 G/L), use of axi-cel and no bridging therapy or SD/PD after bridging. Patients with a CRS-PSS score > 2 had significantly higher risk to develop grade ≥ 3 ICANS. Both scores were externally validated in international cohorts of patients treated with tisa-cel or axi-cel.

**Supplementary Information:**

The online version contains supplementary material available at 10.1186/s13045-024-01579-w.

## Purpose

Chimeric antigen receptor (CAR) T cells directed against the CD19 antigen have emerged as one of the most potent treatments for relapsed/refractory (R/R) large B-cell lymphoma (LBCL) [1–9]. Axicabtagene ciloleucel (axi-cel) and lisocabtagene maraleucel (liso-cel) are now approved for second-line or subsequent lines of treatment, while tisagenlecleucel (tisa-cel) is approved after at least 2 previous lines. However, CAR T cells are associated with some early-onset specific toxicities, such as cytokine release syndrome (CRS) and immune effector cell-associated neurotoxicity (ICANS), which can be life-threatening [10–12]. Moreover, severe (i.e. grade ≥ 3) CRS and ICANS can lead to intensive care unit (ICU) admission in up to 30% of these patients, significantly prolong hospitalization, and add to the already significant cost of treatment [13].

Recently, many real-world evidence (RWE) studies have confirmed similar efficacy as in trials [14–20]. Grade ≥ 3 CRS still occurs in real life in approximately 5–15% of patients regardless of the CAR T product (axi-cel or tisa-cel), and grade ≥ 3 ICANS occurs in 15–40% of patients treated with axi-cel compared with approximately 5–15% of patients treated with tisa-cel.

Several attempts to discover robust predictors of severe CRS or ICANS have been made [15, 16, 19, 20]. The early identification of patients at high risk of severe toxicity has become of utmost importance now that CAR T cells are broadly used in routine practice and are still associated with significant morbidity, medical costs and complex patient flow [1, 10–18].

Several scoring systems have been proposed to predict the risk of CRS or ICANS. The m-EASIX (modified Endothelial Activation and Stress Index) and the s-EASIX (simplified EASIX) based on the EASIX score designed for graft-versus-host disease prediction have been proposed to identify patients who subsequently develop severe CRS or ICANS [21, 22]. In the present study, we report on the specific toxicities of CAR T cells (i.e., CRS and ICANS) in a large RWE patient population treated with axi-cel or tisa-cel for R/R LBCL from the French DESCAR-T registry, retrospectively capturing exhaustive data for all patients treated with CAR T cells in France. We propose two externally validated prognostic scoring systems (PSSs) to refine the identification of patients at low or high risk of severe CRS or ICANS before any CAR T-cell infusion.

## Patients and methods

### Study design and patients

All patients treated in France with axi-cel or tisa-cel from December 2019 to April 2022 and included in the DESCAR-T registry were considered. Data were exported from the registry in May 2022. All patients with LBCL for whom CAR T-cell therapy with tisa-cel or axi-cel was infused in the setting of the first European Medicines Agency (EMA) approval label (i.e., after at least 2 prior lines of treatment) were considered. The protocol was approved by national ethic committee and the data protection agency, and the study was undertaken in accordance with the Declaration of Helsinki. DESCAR-T is registered under the ClinicalTrials.gov identifier NCT04328298. The study was sponsored by the Lymphoma Academic Research Organization (LYSARC).

### External validation patient cohorts

Individual patient data from 3 previously published cohorts from Spain, the United Kingdom (UK), Germany and the United States (US) were extracted and served as an external international validation series [18, 19, 23–25]. The characteristics of patients in each cohort are presented in the corresponding initial publication [18, 19, 23]. A patient flow diagram is presented in Supplementary Figure S1. Definition of bulky disease remains variable in hematology. Tumor diameters from 5 to 10 cm were used in different clinical trials. Of note, the cutoff for bulky disease was set at 5 cm in the training and internal validation cohorts from the DESCAR-T registry, while it was 7 cm in the Spanish dataset, and 10 cm in the UK as well as in the joint dataset from Germany and the US. Since the longest diameter of the largest node or mass was not captured as a continuous parameter in these datasets, recalculation with a 5 cm cutoff could not be performed, and bulky disease was therefore considered in the external validation set with different cutoffs.

### Outcomes

Response was assessed according to the Lugano 2014 criteria based on ^18^fluoro-deoxyglucose positron emission tomography (FDG-PET) after CAR T-cell infusion [26]. FDG-PET was performed at least before lymphodepletion and after 1, 3, 6, 9 and 12 months for all patients according to follow-up duration. For all survival analyses, a landmark time was set at 28 days after CAR T-cell infusion to assess the prognostic impact of CRS and ICANS on outcome. PFS was defined from the landmark time to the date of first documented relapse, progressive disease, date of last follow-up or death from any cause, whichever came first. Overall survival (OS) was defined from the landmark time to the date of death from any cause or the date of last follow-up. CRS and ICANS were graded according to the consensus criteria from the American Society for Transplantation and Cellular Therapy (ASTCT) [4].

### Statistical methods

For PSS computation, the dataset was split into a training set (60% randomly selected, N = 555) to derive optimal predictive models and an internal validation set (the remaining 40% of records, N = 370) to test the validity of the selected models. In the training set, the predictive value of each variable was assessed by 1000 bootstrap replications performing univariable logistic regressions for each toxicity outcome (i.e., grade ≥ 3 CRS or ICANS). Variables that were found to be significant (*P* < 0.05) in at least 50% of the replication sets were eligible for inclusion in multivariable analyses. This approach was applied to select the most consistently predictive parameters. Multivariable analyses were performed following stepwise selection (entry-level *P* = 0.1, retain level *P* = 0.05) in 1000 bootstrap replications for each toxicity endpoint. Based on the multivariable model most frequently selected via the bootstrap procedure above, a simplified risk score was calculated using the rounded median parameter estimates of the bootstrap replications for grade ≥ 3 CRS and ICANS  [27]. The optimal cutoff for risk score dichotomization was considered based on the receiver operating characteristic (ROC) curve and was selected using the value that maximized the Youden's index (J = sensitivity + specificity − 1), defined as the overall correct classification rate minus 1 at the considered cutoff point. No imputation was performed for missing data.

Regarding previously validated predictive scores for CRS and ICANS in the literature, the EASIX score (LDH*creatinine/platelets), the modified EASIX score (m-EASIX: CRP*creatinine/platelets) and the simplified EASIX score (s-EASIX: LDH/platelets) were assessed in our cohort, and the performance of each was compared in both the training and internal validation sets using the AUC of the ROC curve [21].

The PSSs were externally validated using an independent cohort of patients combining data from the UK, Germany, Spain and the US. Overall, data from 725 and 760 patients were available for CRS and ICANS prediction score computation, respectively. Fisher’s exact test or χ^2^ test were used when appropriate for comparing CRS and ICANS incidences according to patient risk category.

Landmark analyses on day 28 were used to assess the prognostic impact of post-infusion parameters (i.e., CRS, ICANS) on subsequent PFS and OS. Survival distributions were compared using the log-rank test. The cumulative incidence of progression and relapse or of non relapse mortality (NRM) was evaluated using competitive risk models, and comparisons between distributions were statistically performed using Gray’s test. A two-sided *P* value of less than 0.05 was considered significant. No adjustment was performed for multiple testing. Survival curves were generated using the Kaplan–Meier estimation method. Statistical analyses were performed using SAS software version 9.4.

## Results

### Patient characteristics and toxicities

Between December 2019 and April 2022, 925 patients from 27 French centers with R/R LBCL after at least two lines of previous therapy underwent a commercial CAR T-cell infusion with axi-cel or tisa-cel treatment and were registered in the French DESCAR-T registry. Patient characteristics are presented in Table 1. Toxicities and their management are presented in Table 2. Tisa-cel was administered in 38% of patients (n = 351), and axi-cel was administered in 62% of patients (n = 574). CRS of any grade occurred in 778 patients (84.1%), with 74 patients (8.0%) with grade 3 CRS or higher. ICANS of any grade occurred in 375 patients (40.5%), with 112 patients (12.1%) experiencing grade ≥ 3 ICANS.

**Table 1:** Patient characteristics in the DESCAR-T cohort (at lymphodepletion)

	DESCAR-T cohort (N = 925) N (%)
Age at time of CAR T-cell infusion (yrs)	
Median (min–max)	63 (18–82)
≥ 65 yrs	401 (43.3)
Sex	
Male	567 (61.3)
Female	358 (38.7)
Histological diagnosis	
De novo aggressive large B-cell lymphoma	
DLBCL NOS or HGBCL	675 (73.8)
PMBCL	42 (4.6)
T/HRLBCL	12 (1.3)
Systemic relapse of PCNSL	5 (0.5)
DLBCL, leg type	5 (0.5)
tFL	135 (14.7)
tMZL	22 (2.4)
Other transformed indolent non-Hodgkin lymphomas	19 (2)
Missing data	10
Number of prior treatment lines	
Median (min;max)	3 (2;10)
≥ 3 prior lines	436 (47.3)
Missing data	3
ECOG PS	
0–1	748 (85.9)
≥ 2	123 (14.1)
Missing data	55
Ann Arbor Stage	
I–II	174 (19.5)
III–IV	717 (80.5)
Missing data	34
aaIPI	
0	61 (7.3)
1	289 (34.5)
2	433 (51.7)
3	54 (6.5)
Missing data	88
Bulk (with a cutoff at 5 cm)	
No	671 (73.2)
Yes	246 (26.8)
Missing	8
Platelets	
< 150 G/L	326 (35.9)
≥ 150 G/L	582 (64.1)
Missing data	18
LDH	
≤ UNL	341 (44.0)
> UNL	434 (56.0)
Missing data	150
CRP	
≤ 30 mg L^−1^	619 (76.2)
> 30 mg L^−1^	193 (23.8)
Missing data	113
Bridging and response to bridging	
No bridging	142 (15.8)
Response to bridging (PR or CR)	249 (27.7)
No response to bridging (SD or PD)	507 (56.4)
Missing data	27

Sum may not equal 100% because of rounding

*aaIPI* age-adjusted international prognostic index, *CR* complete response, *DLBCL* diffuse large B-cell lymphoma, *ECOG* Eastern Cooperative Oncology Group, *LDH* lactate dehydrogenase, *NA* not applicable, *PMBCL* primary mediastinal B-cell lymphoma, *PD* progressive disease, *PCNSL* primary central nervous system lymphoma, *PR* partial response, *PS* performance status, *SD* stable disease, *T/HRLBCL* T-cell/histiocyte-rich large B-cell lymphoma, *tFL* transformed follicular lymphoma, *tMZL* transformed marginal zone lymphoma, *UNL* upper normal limit, *yrs* years

**Table 2:** Toxicity after anti-CD19 CAR T-cell infusion

	All patients (N = 925) N (%)	Tisa-cel (N = 351) N (%)	Axi-cel (N = 574) N (%)
CRS			
All grades	778 (84.1)	266 (75.8)	512 (89)
Grade ≥ 3	74 (8.1)	25 (7.1)	49 (8.5)
Grade 5	5 (0.5)	4 (1.1)	1 (0.2)
Median time to onset—days (IQR)	2 (1–4)	2 (1–3)	3 (1–4)
Median time to resolution—days (IQR)	6 (4–9)	5 (4–7)	6 (4–9)
Missing data	3	1	2
ICANS			
All grades	375 (40.5)	77 (21.9)	298 (51.8)
Grade ≥ 3	112 (12.1)	10 (2.8)	102 (17.8)
Grade 5	2 (0.3)	0 (0)	2 (0.3)
Median time to onset—days (IQR)	6 (4–9)	5 (3–6)	6 (5–9)
Median time to resolution—days (IQR)	6.5 (4–11)	6 (3–9)	7 (4–11)
Missing data	3	1	2
Tocilizumab use (anti-IL-6 receptor)	548 (59.2)	170 (48.4)	378 (65.8)
Median dose tocilizumab—mg (IQR)	983 (600–1614)	800 (582–1388)	1104 (600–1800)
Median duration—days (IQR)	2 (1–3)	2 (1–2)	2 (1–3)
Steroids use (dexamethasone equivalent)	386 (41.7)	100 (28.5)	286 (49.8)
Median dose steroids—mg (IQR)	120 (40–230)	49 (20–170)	125 (40–237)
Median duration—days (IQR)	6 (3–10)	6 (2–9)	6 (4–10)
Anakinra use (anti IL-1)	36 (3.9)	4 (1.1)	32 (5.6)
Median dose anakinra—mg (IQR)	600 (200–999)	450 (100–800)	600 (200–1000)
Median duration—days (IQR)	7 (4–10)	7 (4–10)	7 (4–9)
Indication for use			
Persistent CRS	6 (16.6)	NA	NA
Persistent ICANS	30 (83)	NA	NA
Siltuximab use (anti IL-6)	22	5	17
Median dose siltuximab—mg (IQR)	880 (600–990)	550 (500–550)	890 (700–1045)
Median duration—days (IQR)	1 (1–1)	1 (1–1)	1 (1–1)
Indication for use			
Persistent CRS	16 (73)	NA	NA
Persistent ICANS	6 (27)	NA	NA
Intensive care unit admission	220 (24.1)	64 (18.4)	156 (27.5)
Mean ICU stay, days (IQR)	2.1 (0–2)	1.7 (0–1)	2.3 (0–3)
Missing data	12	4	8

Toxicities were graded according to CTCAE version 5.0 for cytopenia and according to the consensus grading from the ASTCT for CRS and ICANS. Only data for patients who experienced at least grade ≥ 1 toxicity are reported in the table

*CRS* cytokine release syndrome, *ICANS* immune effector cell-associated neurotoxicity syndrome, *ICU* intensive care unit, *IQR* interquartile range, *NA* not available

### Survival according to CRS or ICANS severity

Toxic mortality related to CRS and ICANS (grade 5) during the first 28 days following CAR T-cell infusion was only reported in 5 patients, all due to CRS (Table 2). Two cases of grade 5 ICANS were recorded, occurring on days 29 and 97 post-infusion (with onset following infusion and worsening over time). No deaths related to CRS occurred after day 28. In a competitive risk analysis, the cumulative incidence of NRM was not statistically different between axi-cel and tisa-cel while the rate of relapse and death due to lymphoma was significantly higher with tisa-cel (*P* < 0.0001, Gray’s test, Supplementary Figure S2A and B).

**Fig. 1:**
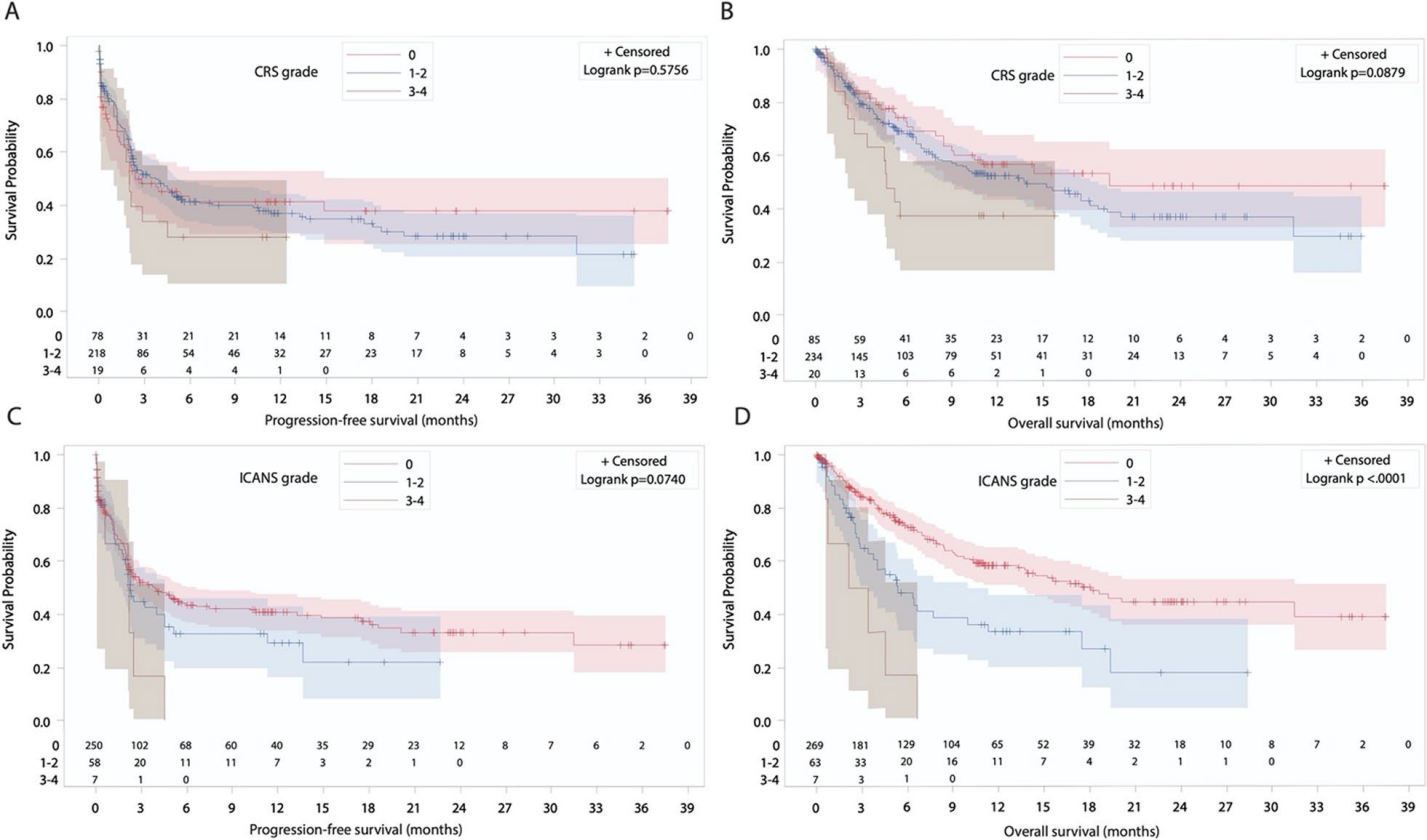
Day 28 landmark survival analysis according to toxicity grade for patients treated with tisa-cel. **A** PFS according to CRS grade. **B** OS according to CRS grade. **C** PFS according to ICANS grade. **D** OS according to ICANS grade

**Fig. 2:**
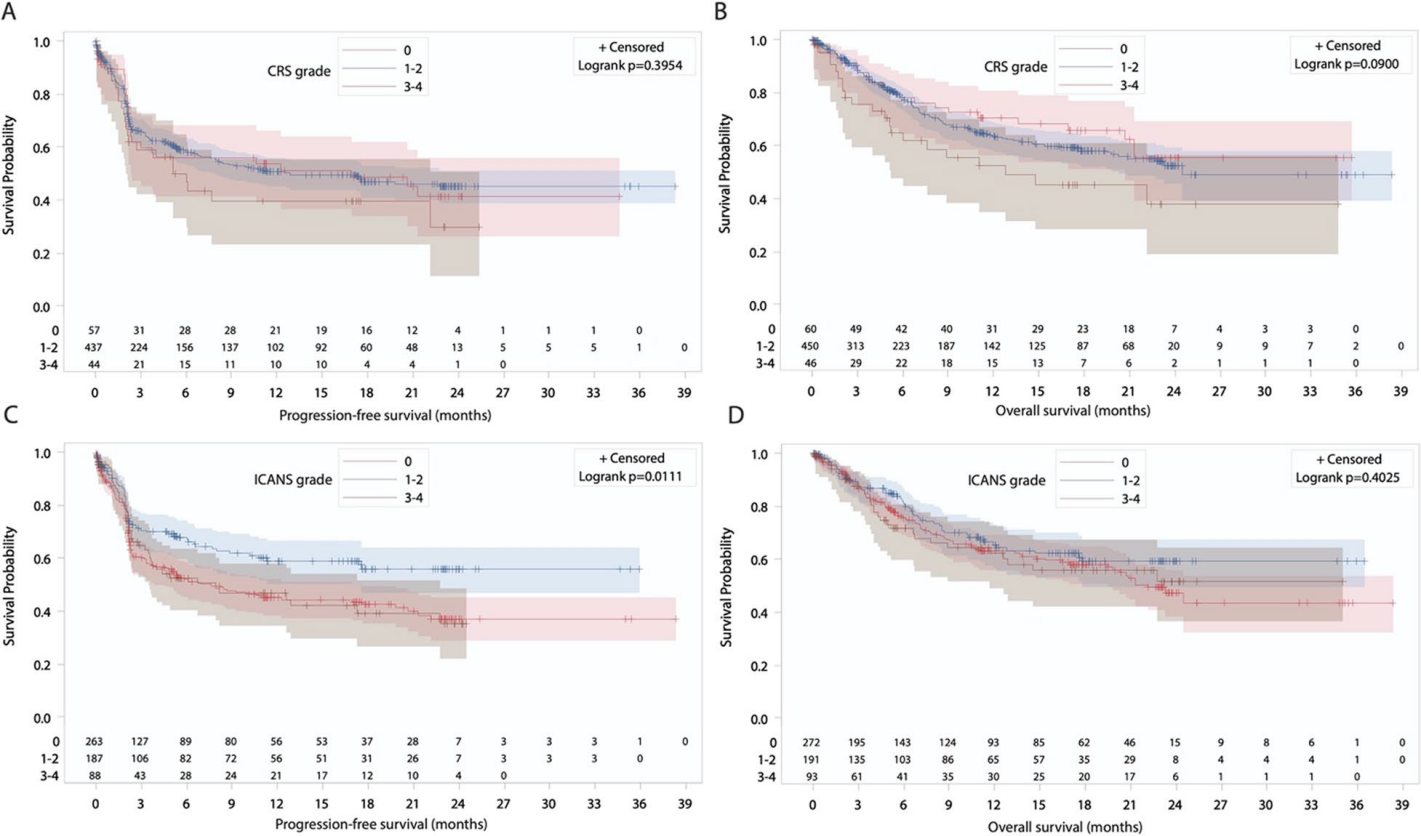
Day 28 landmark survival analysis according to toxicity grade for patients treated with axi-cel. **A** PFS according to CRS grade. **B** OS according to CRS grade. **C** PFS according to ICANS grade. **D** OS according to ICANS grade

The prognostic significance of CRS and ICANS severity on subsequent PFS and OS was analyzed using a 28-day landmark time according to each CAR T product. For patients treated with tisa-cel, no significant impact of CRS severity on PFS or OS was observed (Fig. 1A, B). While no significant association was observed between ICANS severity and PFS, a direct and highly significant correlation between ICANS grade and OS was seen (*P* < 0001, Fig. 1C, D). For axi-cel, patients who experienced mild (grade 1–2) ICANS showed significantly prolonged PFS compared with patients without or with severe ICANS (*P* = 0.011, Fig. 2C) due to a lower cumulative incidence of progression or death due to lymphoma with no NRM difference (Supplementary Figure S3A and B). No OS difference according to ICANS severity was observed (Fig. 2D). Significant associations (i.e. worse OS in case of moderate or severe ICANS for tisa-cel and improved PFS for moderate ICANS for axi-cel) were maintained when considering multivariable models taking into account potential confounding parameters (Supplementary Table S1).

In sensitivity analyses, subsequent outcome after day 28 were similar for patients experiencing CRS or ICANS grade 1 or grade 2 whatever the CAR T received (axi-cel or tisa-cel) or the survival endpoint (PFS or OS) (Supplementary Figures S4 and S5).

### Prognostic analysis of toxicity and scoring systems

To build PSS for grade ≥ 3 CRS and ICANS, the cohort was randomly split into a (60%) training set and a (40%) validation set. No statistically significant differences were observed between the training and validation sets regarding toxicity outcomes or patient characteristics (Supplementary Tables S2 and S3). All biological parameters were considered at lymphodepletion. For CRS, in univariable analyses and when using a bootstrap approach, bulky disease with a largest node or mass > 5 cm, a CRP level > 30 mg/L, a lactate dehydrogenase (LDH) level > 2 times the upper limit of normal (ULN), and a platelet count < 150 G/L were significantly associated with a higher risk of grade ≥ 3 CRS (Supplementary Table S4). In contrast, achieving a complete response (CR) or a partial response (PR) after bridging was predictive of a decreased risk of grade ≥ 3 CRS (compared with patients who did not receive any bridging therapy or those with stable disease (SD) or progressive disease (PD) after bridging). For ICANS, the female sex, the use of axi-cel and a platelet count < 150 G/L were significantly associated with grade ≥ 3 ICANS (Supplementary Table S5). Achieving a CR or a PR after bridging was also predictive of a decreased risk of grade ≥ 3 ICANS.

In multivariable analyses, based on parameters that were most frequently selected by bootstrap analysis, bulky disease, a platelet count < 150 G/L and a CRP level > 30 mg/L were significantly associated with a higher risk of grade ≥ 3 CRS, while achieving a CR or a PR after bridging (compared with no bridging therapy or SD/PD after bridging) was predictive of a decreased risk (Supplementary Table S6). All parameters selected in the univariable analysis were retained in the multivariable analysis for the prediction of grade ≥ 3 ICANS (female sex, platelets < 150 G/L, use of axi-cel and response after bridging) (Supplementary Table S7).

Based on the parameters selected and the associated weighted coefficients by multivariable analyses, two independent PSSs were derived, one for grade ≥ 3 CRS and one for grade ≥ 3 ICANS, and were termed CRS-PSS (4-point scale) and ICANS-PSS (5-point scale), respectively (Table 3). Each score was subsequently divided into 2 classes for convenient routine use with an optimal cutoff set at 2 (value that maximized the Youden’s index). For severe CRS, the incidence was 5.9% in the low-risk category (i.e., CRS-PSS ≤ 2) compared with 19.8% in the high-risk category (i.e., CRS-PSS > 2). For severe ICANS, the incidence was 2.6% in the low-risk category (i.e., ICANS-PSS ≤ 2) compared with 18.3% in the high-risk category (i.e., ICANS-PSS > 2). While positive predictive values (PPVs) for both CRS- and ICANS-PSS did not exceed 20%, high negative predictive values (NPVs) of more than 95% were achieved for both scoring systems. The statistical prognostic significance of both CRS-PSS and ICANS-PSS was confirmed in the DESCAR-T internal validation cohort (Table 3). CRS-PSS and ICANS-PSS showed consistently better performances with higher AUC of the ROC curve than the EASIX, m-EASIX and s-EASIX in the validation cohort (Supplementary Table S8).

**Table 3:** CRS-PSS (prognostic scoring system) and ICANS-PSS in the training and validation sets

	Factors and score computation^a^		Category	n/N (%) of grade ≥ 3 AE^b^ (CRS for CRS-PSS and ICANS for ICANS-PSS)		
				Training set^c^ (N = 533)	DESCAR-T validation set^d^ (N = 351)	External validation set^e^ (N = 725)
CRS-PSS 4 points	Bulk (> 5 cm)	+ 1	Low (0–2)	26/442 (5.9%)	15/283 (5.3%)	33/549 (6.0%)
	Platelets < 150 G/L	+ 1				
	No bridge or bridge failure^f^	+ 1	High (> 2)	18/91 (19.8%)	9/68 (13.2%)	26/176 (14.8%)
	CRP > 30 mg/L	+ 1				

			Category	Training set^c^ (N = 554)	DESCAR-T validation set^d^ (N = 369)	External validation set^e^ (N = 760)
ICANS-PSS 5 points	Female sex	+ 1	Low (0–2)	6/232 (2.6%)	5/149 (3.3%)	13/299 (4.3%)
	Platelets < 150 G/L	+ 1				
	No bridge or bridge failure^f^	+ 1	High (> 2)	59/322 (18.3%)	40/220 (18.2%)	88/461 (19.1%)
	Axi-cel	+ 2				

^a^At lymphodepletion

^b^Numbers of patients differ between the CRS-PSS and ICANS-PSS because of various missing parameters between the 2 scores

^c^*P* < 0.0001 for both CRS-PSS and ICANS-PSS

^d^*P* = 0.030 for CRS-PSS and *P* < 0.001 for ICANS-PSS

^e^Aggregated retrospective data from Spain, Germany, UK and US (See Supplementary Table S8). *P* < 0.001 for CRS-PSS and *P* < 0.001 for ICANS-PSS

^f^Bridge failure is defined by a stable or progressive disease after bridging

The two scoring systems were then externally validated in an international set of patients from previously published series in Spain, the UK, the US and Germany (Table 3, Supplementary Figure S1 and Supplementary Table S9) [18, 19, 23–25]. In total, data for score computation were available for 725 and 760 patients for CRS-PSS and ICANS-PSS, respectively. In this external validation set, 6.0% of patients with a low CRS-PSS score developed severe CRS compared with 14.8% of those with a high CRS-PSS score (*P* < 0.001). Regarding ICANS, 4.3% and 19.1% of patients in the low- and high-risk groups, respectively, developed severe toxicity (*P* < 0.001).

## Discussion

Anti-CD19 CAR T cells have dramatically altered the therapeutic armamentarium and the prognosis of patients with R/R LBCL in the last few years [4–9]. Despite notable improvement in toxicity management following early mitigation strategies with anti-IL6R and steroids, CRS and ICANS, two specific side effects, are still the leading causes of acute morbidity, ICU transfer and prolonged hospitalization [10, 13]. In this multicenter RWE study based on the French DESCAR-T registry encompassing nearly a thousand patients treated with commercial tisa-cel or axi-cel after at least 2 lines of treatment, we identified several parameters associated with grade ≥ 3 CRS or ICANS. As expected, bulky or uncontrolled disease before lymphodepletion and a high LDH or CRP level were associated with a significantly more frequent incidence of grade ≥ 3 CRS. Moreover, a platelet count below 150 G/L, already identified in the context of graft-versus-host disease and whose validity has been confirmed by others in predicting severe CRS, was indeed found to be significantly associated with severe CRS in our series [21, 22]. The absence of a response following bridging therapy and a low platelet count were associated with grade ≥ 3 ICANS as well. Surprisingly, the absence of bridging therapy was also associated with a significantly higher risk of severe CRS and/or ICANS, similar to stable or progressive disease after bridging, indicating that bridging therapy could limit severe toxicity following infusion by limiting tumor burden progression or by another mechanism that has yet to be identified. Other recent reports have found an increased risk of any-grade ICANS in the absence of response to bridging therapy or in case of untreated relapse [28, 29]. It is intriguing given that observed toxicity following axi-cel treatment in real-world data, where bridging is largely used, is indeed found at a much lower rate than in pivotal trials in which only corticosteroids were allowed. As expected, the most predictive parameter for severe ICANS was the use of axi-cel compared with tisa-cel. Unexpectedly, the female sex was robustly associated with severe ICANS. Such an observation was also of borderline significance in the univariable analysis in a study by Nastoupil and colleagues considering patients treated with axi-cel [15]. Of note, ferritin levels are not abstracted in the DESCAR-T registry and were not assessable for use in the prognostic models. Based on independent prognostic parameters, two scoring systems were built and robustly identified patients with a higher risk of grade ≥ 3 CRS or ICANS. The two scoring systems were found to be more discriminant than the previously proposed EASIX, modified EASIX and simplified EASIX scoring systems. Whether the 2 scoring systems will remain valid in the 2nd line setting and considering liso-cel instead of tisa-cel (associated with a similarly low rate of severe toxicity) needs to be confirmed. We acknowledge that retrospective data collection might have led to specific biases compared to prospective trials. It must also be recognized that even in the high-risk categories, only 15–20% of patients experienced grade ≥ 3 CRS and ICANS in the training and validation cohorts. This is reflected by the high NPV but limited PPV of the scoring systems, consistent with other predictive models of CAR T-cell toxicity [30]. However, from the perspective of potential future outpatient CAR T-cell infusions, the NPV would prevail over the PPV. This also highlights how a substantial number of biological and intrinsic features of CAR T-cell products associated with severe toxicity are likely not fully captured by baseline patient and disease characteristics. The cut-off was set at 2 due to the choice of the best trade-off between identifying most patients that could be managed on an outpatient setting (with low-score risk) and increasing the population that could benefit from the use of early mitigation strategies like tocilizumab and dexamethasone (in case of high-risk score). Depending on the clinical context, physician could use a higher cut-off above 2 for increasing PPV for instance. Another limitation of the present work is the different cutoffs used in the training and the external validation sets for bulk definition. Various cutoffs were used throughout different nationwide registries and continuous measurement was not captured to allow for retrospective computation. However, a cutoff set at 5 cm was internally validated in the DESCAR-T registry and marginal differences were observed in the external validation set with comparable patient repartition in the low- and high-risk categories. We advocate for using a 5 cm cutoff for bulk definition for score computation, but 7.5 cm and 10 cm would likely perform similarly at a population level.

Divergent data exist regarding the impact of acute toxicity and therapeutic intervention on subsequent outcomes [19, 31–34]. The incidence of grade 5 CRS or ICANS was extremely low in the present cohort. Interestingly, in the 28-day landmark analyses, divergent prognostic associations with PFS and OS were observed according to CAR T-cell product. ICANS severity had a major impact on OS in patients treated with tisa-cel, while no difference was observed in those treated with axi-cel. Surprisingly, patients treated with axi-cel presenting low-grade (1–2) ICANS had a significantly prolonged PFS compared with patients experiencing no or severe neurotoxicity. This could reflect a higher CAR T-cell proliferation peak, in line with previous reports showing better disease control in cases of low-grade toxicity [33, 34]. In addition to similar patient management for grade 1 or 2 CRS and ICANS without usual need for ICU transfer, subsequent PFS and OS were also comparable justifying grouping grade 1 and 2 versus 3 and 4 for prognostic scoring development in the study.

In conclusion, our study provides RWE estimates of CRS and ICANS incidence and severity, as well as the impact of toxicities on subsequent outcomes based on a large cohort of patients. We propose two validated and easy-to-use preinfusion scoring systems that allow for the identification of patients at very low risk of severe CRS or ICANS for tailored medical management.

## Supplementary Information

Supplementary Material 1.

## Data Availability

All data generated or analyzed during this study are included in this published article and its supplementary information file.
