# Supplementary material for: Novel prognostic scoring systems for severe CRS and ICANS after anti-CD19 CAR T cells in large B-cell lymphoma
Source: J Hematol Oncol. 2024 Aug 6;17:61. doi: 10.1186/s13045-024-01579-w (PMC11305039; doi:10.1186/s13045-024-01579-w)

**Supplementary Table 1. Multivariable models**

| Label | Modality | Hazard Ratio | 95% Hazard Ratio Confidence Limits | | P value |
| --- | --- | --- | --- | --- | --- |
|  |  |  | Lower | Upper |  |
| Multivariable model for OS according to ICANS grade in patients treated with tisa-cel | | | | | |
| ICANS grade* | 1-2 | 1.785 | 1.166 | 2.731 | 0.007 |
|  | 3-4 | 3.168 | 1.222 | 8.214 | 0.017 |
| Bulk >5cm | Yes | 1.584 | 1.071 | 2.344 | 0.021 |
| LDH elevated | Yes | 2.274 | 1.514 | 3.416 | <.001 |
| Multivariable model for PFS according to ICANS grade in patients treated with axi-cel | | | | | |
| ICANS grade* | 1-2 | 0.672 | 0.485 | 0.932 | 0.017 |
|  | 3-4 | 0.785 | 0.526 | 1.170 | 0.234 |
| Bulk >5cm | Yes | 1.486 | 1.100 | 2.007 | 0.009 |
| CRP > 30mg | Yes | 1.605 | 1.158 | 2.227 | 0.004 |
| Stage | III-IV | 1.645 | 1.117 | 2.423 | 0.011 |

*reference category: no ICANS.

In all models, effect of ICANS on survival were adjusted for the following parameters: number of prior lines (≤3 v >3), age (≤65 v > 65 years), bulky disease (≤5 v > 5cm), CRP (≤30 v > 30mg/L), sex, stage (I-II v III-IV), ECOG (≤2 v >2) and LDH (≤ULN v > ULN).

**Supplementary Table 2. Baseline patient characteristics between randomly selected training and validation sets from the DESCAR-T registry for toxicity scoring systems building**

|  | Validation Set | | Training Set | | All | | P value |
| --- | --- | --- | --- | --- | --- | --- | --- |
|  | N=370 | | N=555 | | N=925 | |  |
| Sex |  |  |  |  |  |  | 0.508 |
| Male | 222 | (60.0%) | 345 | (62.2%) | 567 | (61.3%) |  |
| Female | 148 | (40.0%) | 210 | (37.8%) | 358 | (38.7%) |  |
| Age≥65 |  |  |  |  |  |  | 0.828 |
| No | 208 | (56.2%) | 316 | (56.9%) | 524 | (56.6%) |  |
| Yes | 162 | (43.8%) | 239 | (43.1%) | 401 | (43.4%) |  |
| Diagnosis group |  |  |  |  |  |  | 1.000 |
| Transformed Indolent | 62 | (16.8%) | 93 | (16.8%) | 155 | (16.8%) |  |
| Aggressive | 308 | (83.2%) | 462 | (83.2%) | 770 | (83.2%) |  |
| At least 3 prior lines |  |  |  |  |  |  | 0.027 |
| No | 180 | (48.6%) | 311 | (56.0%) | 491 | (53.1%) |  |
| Yes | 190 | (51.4%) | 244 | (44.0%) | 434 | (46.9%) |  |
| Ann Arbor stage |  |  |  |  |  |  | 0.126 |
| I-II | 78 | (21.1%) | 96 | (17.3%) | 174 | (18.8%) |  |
| III-IV | 276 | (74.6%) | 441 | (79.5%) | 717 | (77.5%) |  |
| Missing | 16 | (4.3%) | 18 | (3.2%) | 34 | (3.7%) |  |
| ECOG in class (LD then INF) |  |  |  |  |  |  | 0.222 |
| 0-1 | 305 | (82.4%) | 443 | (79.8%) | 748 | (80.9%) |  |
| ≥2 | 43 | (11.6%) | 80 | (14.4%) | 123 | (13.3%) |  |
| Missing | 22 | (5.9%) | 32 | (5.8%) | 54 | (5.8%) |  |
| Bulk |  |  |  |  |  |  | 0.128 |
| No | 260 | (70.3%) | 411 | (74.1%) | 671 | (72.5%) |  |
| Yes | 109 | (29.5%) | 137 | (24.7%) | 246 | (26.6%) |  |
| Missing | 1 | (0.3%) | 7 | (1.3%) | 8 | (0.9%) |  |
| CR/PR prior to CAR-T |  |  |  |  |  |  | 0.770 |
| No | 260 | (70.3%) | 385 | (69.4%) | 645 | (69.7%) |  |
| Yes | 110 | (29.7%) | 170 | (30.6%) | 280 | (30.3%) |  |
| Bridging class |  |  |  |  |  |  | 0.230 |
| No bridge | 66 | (17.8%) | 76 | (13.7%) | 142 | (15.4%) |  |
| Bridge CR/PR | 97 | (26.2%) | 152 | (27.4%) | 249 | (26.9%) |  |
| Bridge SD/PD | 207 | (55.9%) | 327 | (58.9%) | 534 | (57.7%) |  |
| CAR-T Name |  |  |  |  |  |  | 0.934 |
| Tisa-cel | 141 | (38.1%) | 210 | (37.8%) | 351 | (37.9%) |  |
| Axi-cel | 229 | (61.9%) | 345 | (62.2%) | 574 | (62.1%) |  |
| CRP > 30 (mg/L) |  |  |  |  |  |  | 0.155 |
| No | 234 | (63.2%) | 385 | (69.4%) | 619 | (66.9%) |  |
| Yes | 84 | (22.7%) | 109 | (19.6%) | 193 | (20.9%) |  |
| Missing | 52 | (14.1%) | 61 | (11.0%) | 113 | (12.2%) |  |
| LDH class |  |  |  |  |  |  | 0.404 |
| Normal | 129 | (34.9%) | 212 | (38.2%) | 341 | (36.9%) |  |
| ULN<-2xULN | 138 | (37.3%) | 206 | (37.1%) | 344 | (37.2%) |  |
| >2xULN | 41 | (11.1%) | 49 | (8.8%) | 90 | (9.7%) |  |
| Missing | 62 | (16.8%) | 88 | (15.9%) | 150 | (16.2%) |  |
| Platelets > 150 (G/L) |  |  |  |  |  |  | 0.583 |
| No | 134 | (36.2%) | 192 | (34.6%) | 326 | (35.2%) |  |
| Yes | 228 | (61.6%) | 353 | (63.6%) | 581 | (62.8%) |  |
| Missing | 8 | (2.2%) | 10 | (1.8%) | 18 | (1.9%) |  |
| Serum creatinine (µmol/L) |  |  |  |  |  |  | 0.770 |
| Normal | 299 | (80.8%) | 453 | (81.6%) | 752 | (81.3%) |  |
| > Upper limit | 49 | (13.2%) | 70 | (12.6%) | 119 | (12.9%) |  |
| Missing | 22 | (5.9%) | 32 | (5.8%) | 54 | (5.8%) |  |
| Hemoglobin ≤ 10.0 (g/dL) |  |  |  |  |  |  | 0.794 |
| No | 207 | (55.9%) | 317 | (57.1%) | 524 | (56.6%) |  |
| Yes | 155 | (41.9%) | 229 | (41.3%) | 384 | (41.5%) |  |
| Missing | 8 | (2.2%) | 9 | (1.6%) | 17 | (1.8%) |  |
| Lymphocytes (ALC) < 0.8 (G/L) |  |  |  |  |  |  | 0.383 |
| No | 107 | (28.9%) | 178 | (32.1%) | 285 | (30.8%) |  |
| Yes | 220 | (59.5%) | 321 | (57.8%) | 541 | (58.5%) |  |
| Missing | 43 | (11.6%) | 56 | (10.1%) | 99 | (10.7%) |  |

**Supplementary Table 3. Toxicity incidence between randomly selected training and validation sets from the DESCAR-T registry for toxicity scoring systems building**

|  | Validation Set | | Training Set | | All | | P value |
| --- | --- | --- | --- | --- | --- | --- | --- |
|  | N=370 | | N=555 | | N=925 | |  |
| CRS grade ≥2 |  |  |  |  |  |  | 0.698 |
| No | 228 | (61.6%) | 349 | (62.9%) | 577 | (62.4%) |  |
| Yes | 142 | (38.4%) | 206 | (37.1%) | 348 | (37.6%) |  |
| CRS grade ≥3 |  |  |  |  |  |  | 0.520 |
| No | 343 | (92.7%) | 508 | (91.5%) | 851 | (92.0%) |  |
| Yes | 27 | (7.3%) | 47 | (8.5%) | 74 | (8.0%) |  |
| ICANS grade ≥2 |  |  |  |  |  |  | 0.849 |
| No | 282 | (76.2%) | 426 | (76.8%) | 708 | (76.5%) |  |
| Yes | 88 | (23.8%) | 129 | (23.2%) | 217 | (23.5%) |  |
| ICANS grade ≥3 |  |  |  |  |  |  | 0.836 |
| No | 325 | (87.8%) | 490 | (88.3%) | 815 | (88.1%) |  |
| Yes | 45 | (12.2%) | 65 | (11.7%) | 110 | (11.9%) |  |

**Supplementary Table 4. Univariate bootstrap analysis for prognosis parameters selection for grade ≥3 CRS (in bold parameters selected in more than 50% of iterations)**

| Predictor | Level | Odds Ratio | Bootstrap 95% CI | | Proportion of bootstrap models where the parameters reach a P<0.05 significance rate |
| --- | --- | --- | --- | --- | --- |
|  |  |  | Lower | Upper |  |
| Sex | Female | 1.02 | 0.50 | 2.05 | 0.068 |
| Age≥65-yr | Yes | 0.72 | 0.34 | 1.40 | 0.199 |
| Histology | De novo | 1.85 | 0.78 | 9.13 | 0.241 |
| Stage | III-IV | 1.09 | 0.53 | 3.15 | 0.059 |
| ECOG | ≥2 | 1.80 | 0.73 | 3.85 | 0.314 |
| **Bulky disease** | **Yes** | **2.90** | **1.50** | **5.52** | **0.898** |
| **CRP>30 mg/L** | **Yes** | **2.88** | **1.41** | **5.80** | **0.859** |
| **LDH>2xULN** | **Yes** | **3.16** | **1.29** | **6.55** | **0.73** |
| **Platelets>150 G/L** | **No** | **2.02** | **1.04** | **3.79** | **0.625** |
| Creatinine | > ULN | 1.32 | 0.44 | 2.84 | 0.105 |
| Hemoglobin ≤ 10 g/dL | Yes | 1.35 | 0.72 | 2.55 | 0.181 |
| Leukocytes < 3.0 G/L | Yes | 0.89 | 0.36 | 1.87 | 0.059 |
| Neutrophils ≤ 1.0 G/L | Yes | 2.01 | 0.73 | 4.33 | 0.347 |
| Lymphocytes < 0.8 G/L | Yes | 1.16 | 0.59 | 2.55 | 0.079 |
| **Bridging** | **Bridge SD/PD** | **7.02** | **2.71** | **784516.0** | **0.98** |
|  | **No bridge** | **4.67** | **0.99** | **577549.3** | **0.98** |
| CAR T product | Axi-cel | 1.08 | 0.60 | 2.09 | 0.057 |

**Supplementary Table 5. Univariate bootstrap analysis for prognosis parameters selection for grade ≥3 ICANS (in bold parameters selected in more than 50% of iterations)**

| Predictor | Level | Odds Ratio | Bootstrap 95% CI | | Proportion of bootstrap models where the parameters reach a P<0.05 significance rate |
| --- | --- | --- | --- | --- | --- |
|  |  |  | Lower | Upper |  |
| **Sex** | **Female** | **2.27** | **1.31** | **3.79** | **0.849** |
| Age≥65-yr | Yes | 1.14 | 0.66 | 1.96 | 0.086 |
| Histology | De novo | 0.63 | 0.34 | 1.23 | 0.289 |
| Stage | III-IV | 1.56 | 0.75 | 4.31 | 0.187 |
| ECOG | ≥2 | 1.31 | 0.56 | 2.55 | 0.118 |
| Bulky disease | Yes | 1.00 | 0.49 | 1.78 | 0.054 |
| CRP>30 mg/L | Yes | 1.36 | 0.62 | 2.44 | 0.165 |
| LDH>2xULN | Yes | 1.81 | 0.69 | 3.75 | 0.296 |
| **Platelets>150 G/L** | **No** | **1.92** | **1.08** | **3.47** | **0.666** |
| Creatinine | > ULN | 1.85 | 0.85 | 3.40 | 0.411 |
| Hemoglobin ≤ 10 g/dL | Yes | 1.19 | 0.71 | 2.05 | 0.109 |
| Leukocytes < 3.0 G/L | Yes | 1.78 | 1.03 | 3.06 | 0.505 |
| Neutrophils ≤ 1.0 G/L | Yes | 1.18 | 0.44 | 2.32 | 0.062 |
| Lymphocytes < 0.8 G/L | Yes | 0.93 | 0.54 | 1.79 | 0.064 |
| **Bridging** | **Bridge SD/PD** | **2.04** | **0.98** | **4.77** | **0.734** |
|  | **No bridge** | **3.44** | **1.47** | **9.44** | **0.734** |
| **CAR T product** | **Axi-cel** | **8.82** | **3.95** | **40.96** | **1** |

**Supplementary Table 6. Parameters finally selected for grade ≥3 CRS based on parameters most frequently selected in 1,000 bootstrap multivariate models incorporating variables retained from univariates analyses**

| Label | Odds Ratio | Wald 95% Confidence Interval | |
| --- | --- | --- | --- |
|  |  | Lower limit | Upper limit |
| Bulky disease (> 5 cm) : Yes vs No | 2.101 | 1.063 | 4.153 |
| CRP>30 mg/L (at lymphodepletion) : Yes vs No | 1.932 | 0.954 | 3.912 |
| Platelets>150 G/L (at lymphodepletion) : No vs Yes | 2.002 | 1.032 | 3.883 |
| Bridging: SD/PD v CR/PR after bridge | 4.179 | 1.221 | 14.306 |
| Bridging: No bridge v CR/PR after bridge | 2.826 | 0.607 | 13.161 |
|  |  |  |  |

**Supplementary Table 7. Parameters finally selected for grade ≥3 ICANS based on parameters most frequently selected in 1,000 bootstrap multivariate models incorporating variables retained from univariates analyses**

| Label | Odds Ratio | Wald 95% Confidence Interval | |
| --- | --- | --- | --- |
|  |  | Lower limit | Upper limit |
| CAR-T: axi-cel v tisa-cel | 8.063 | 3.159 | 20.579 |
| Sex : female vs male | 1.957 | 1.130 | 3.388 |
| Platelets >150 G/L (at lymphodepletion) : No vs Yes | 1.977 | 1.139 | 3.432 |
| Bridging: SD/PD v CR/PR after bridge | 1.824 | 0.866 | 3.841 |
| Bridging: No bridge v CR/PR after bridge | 3.101 | 1.264 | 7.608 |
|  |  |  |  |

**Supplementary Table 8. Model performance comparisons**

|  |  | Training set | DESCAR-T validation set |
| --- | --- | --- | --- |
|  | Score | AUC of the ROC curve  (95% Wald confidence limits) | AUC of the ROC curve  (95% Wald confidence limits) |
| Grade ≥3 CRS prediction | CRS-PSS | 0.72 (0.65-0.79) | 0.61 (0.50-0.72) |
|  | EASIX | 0.64 (0.55-0.73) | 0.57 (0.46-0.69) |
|  | Modified-EASIX | 0.62 (0.52-0.73) | 0.61 (0.48-0.73) |
|  | Simplified-EASIX | 0.63 (0.54-0.73) | 0.57 (0.46-0.69) |
| Grade ≥3 ICANS prediction | ICANS-PSS | 0.75 (0.70-0.80) | 0.71 (0.64-0.78) |
|  | EASIX | 0.60 (0.52-0.67) | 0.56 (0.44-0.67) |
|  | Modified-EASIX | 0.53 (0.44-0.62) | 0.61 (0.41-0.73) |
|  | Simplified-EASIX | 0.58 (0.50-0.65) | 0.58 (0.47-0.69) |

**Supplementary Table 9. International external validation cohorts**

|  |  | n/N (%) of grade ≥3 AE | | | |
| --- | --- | --- | --- | --- | --- |
|  | Category | UK cohort  (N=275) | Spanish cohort  (N=267) | German/US cohort (Munich+Moffitt)  (N=183) |  |
| CRS-PSS  4 points | Low (0-2) | 14/226 (6.2%) | 8/166 (4.8%) | 11/157 (7.0%) |  |
|  | High (>2) | 7/49 (14.3%) | 11/101 (10.9%) | 8/26 (30.8%) |  |
|  |  | UK cohort  (N=295) | Spanish cohort  (N=267) | German/US cohort (Munich+Moffitt)  (N=198) |  |
| ICANS-PSS  5 points | Low (0-2) | 5/93 (5.4%) | 5/125 (4.0%) | 3/81 (3.7%) |  |
|  | High (>2) | 41/202 (20.3%) | 24/142 (16.9%) | 33/117 (28.2%) |  |

**Supplementary Figure Legends**

**Supplementary Figure 1.** Patient flow.

^1^Kuhnl A, Roddie C, Kirkwood AA, et al. A national service for delivering CD19 CAR-T in large B-cell lymphoma - The UK real-world experience. Br J Haematol. 2022;198(3):492-502.

^2^Rejeski K, Perez A, Iacoboni G, et al. The CAR-HEMATOTOX risk-stratifies patients for severe infections and disease progression after CD19 CAR-T in R/R LBCL. J Immunother Cancer. 2022;10(5).

^3^Won M, Iacoboni G, Reguera JL, et al. Axicabtagene ciloleucel compared to tisagenlecleucel for the treatment of aggressive B-cell lymphoma. Haematologica. 2022.

**Supplementary Figure 2**. A. Cumulative incidence of NRM according to CAR T product. B. Cumulative incidence of relapse and death due to lymphoma according to CAR T product.

**Supplementary Figure 3**. A. Cumulative incidence of progression / lymphoma death by ICANS grade. B. Cumulative incidence of death without progression by ICANS grade

**Supplementary Figure 4.** Day 28 landmark survival of Tisa-cel patients according to CRS and ICANS . A. PFS according to CRS grade. B. PFS according to ICANS grade. C. OS according to CRS grade. B. OS according to ICANS grade.

**Supplementary Figure 5.** Day 28 landmark survival of Axi-cel patients according to CRS and ICANS . A. PFS according to CRS grade. B. PFS according to ICANS grade. C. OS according to CRS grade. B. OS according to ICANS grade.

**Supplementary Figure 1.**

**Supplementary Figure 2.**


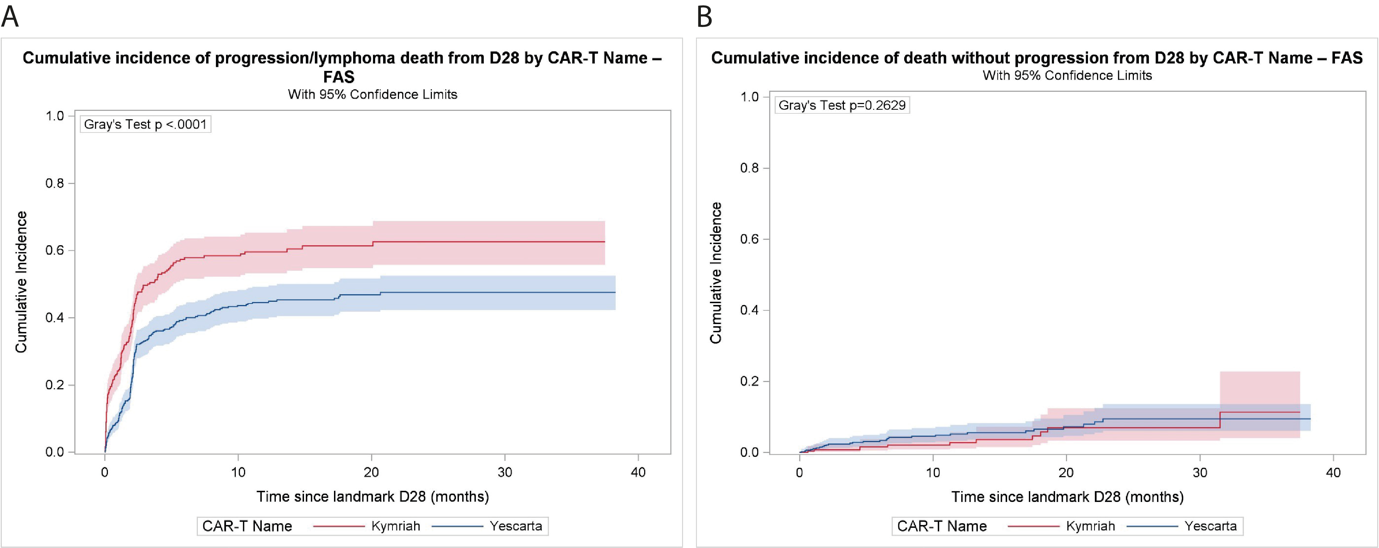


**Supplementary Figure 3.**


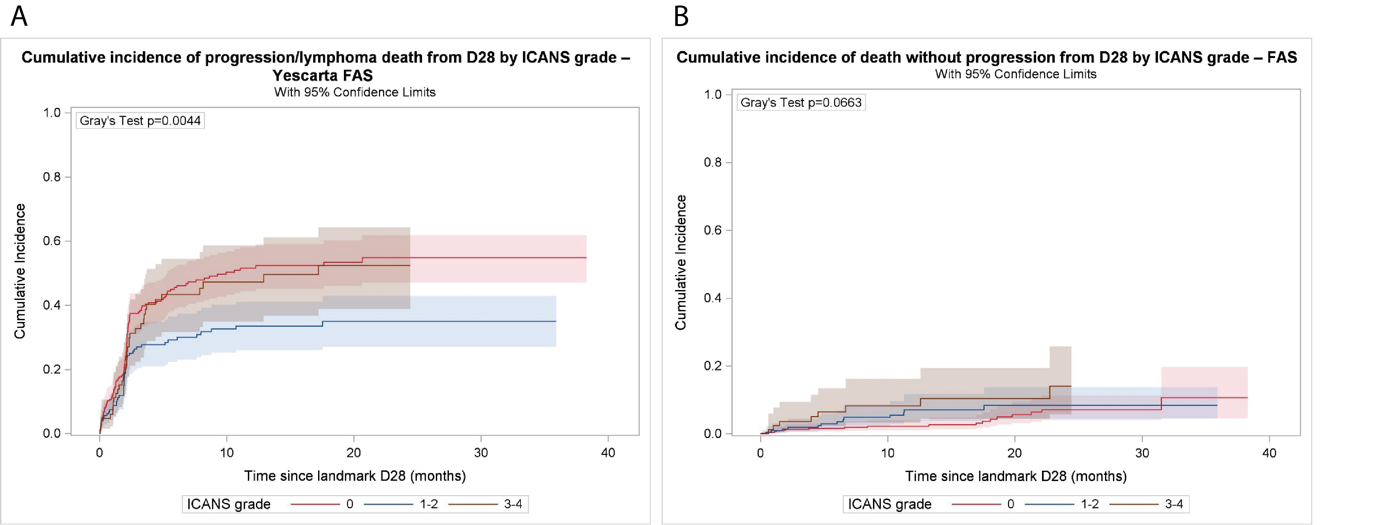


**Supplementary Figure 4.**


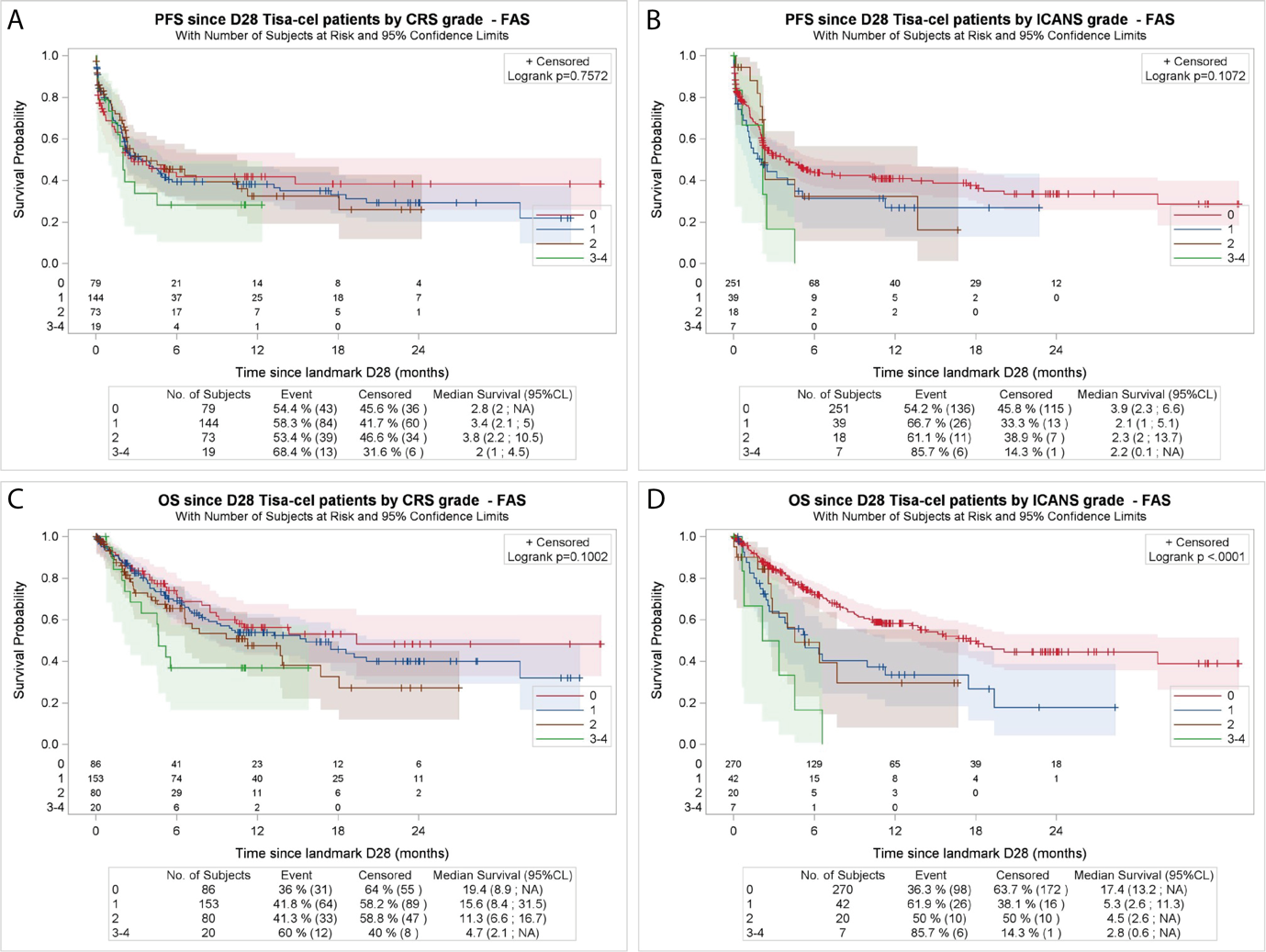


**Supplementary Figure 5.**


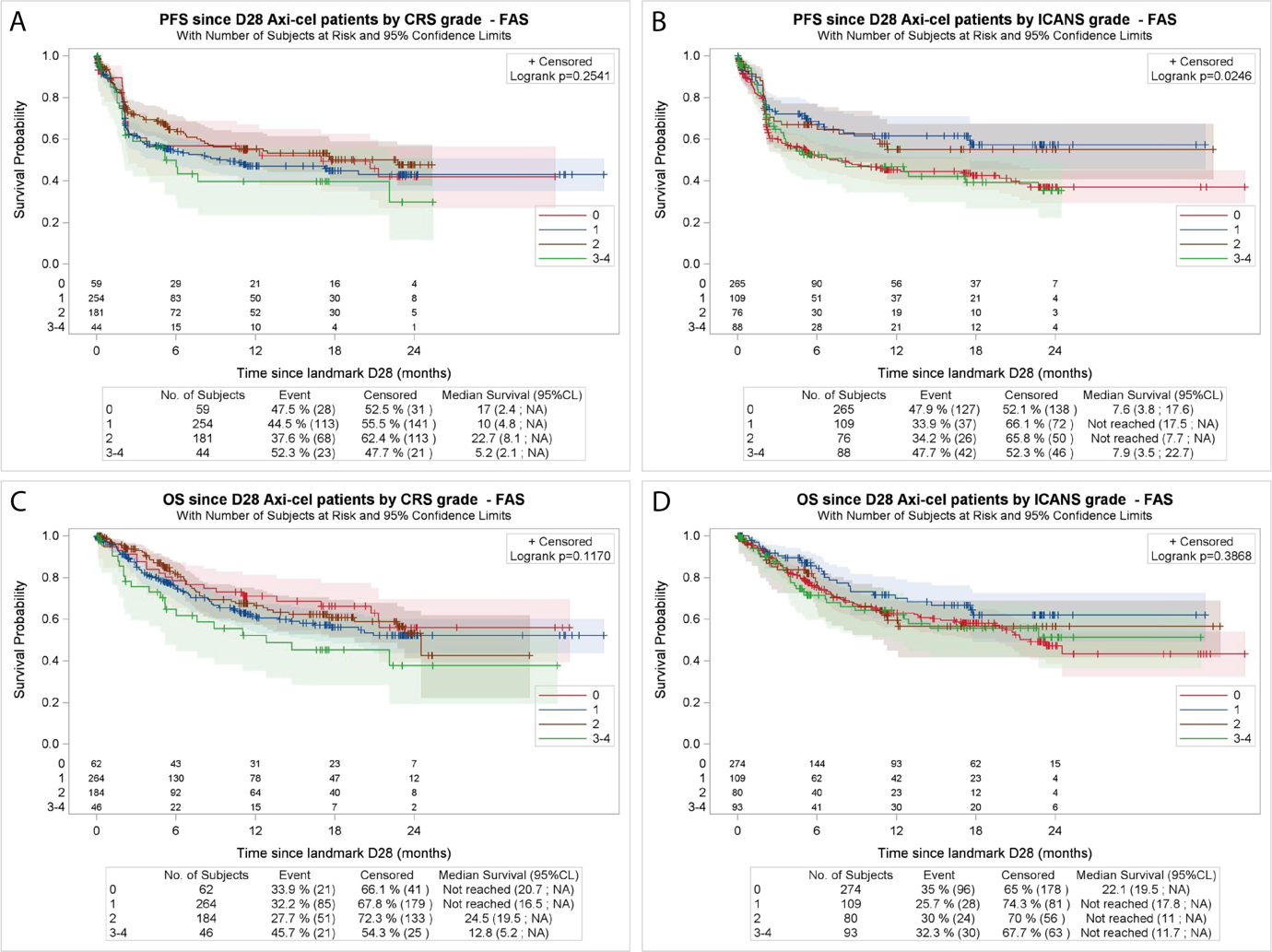

Supplement: Supplementary file 1 — Supplementary Material 1. [file 13045_2024_1579_MOESM1_ESM.docx]
